# Ketogenic Diet in Therapy and Rehabilitation

**DOI:** 10.3390/nu17172855

**Published:** 2025-09-03

**Authors:** Antonio Paoli, Antonino Bianco, Tatiana Moro

**Affiliations:** 1 Department of Biomedical Sciences, University of Padova, 35131 Padua, Italy; tatiana.moro@unipd.it; 2 Sport and Exercise Sciences Research Unit, University of Palermo, 90128 Palermo, Italy; antonino.bianco@unipa.it

The ketogenic diet (KD), long established as an effective medical nutrition therapy for drug-resistant epilepsy, has recently re-emerged as a versatile intervention across multiple therapeutic and rehabilitative domains. Characterized by a high-fat, very low-carbohydrate macronutrient composition, KD induces sustained ketosis with inherent metabolic and signaling effects that extend well beyond seizure control. This Special Issue, “Ketogenic Diet in Therapy and Rehabilitation”, curates contemporary studies that probe KD’s evolving role in neuroprotection, metabolic regulation, oncology, psychiatric care, and functional recovery. KD’s clinical journey began in the 1920s as a dietary approach to mimic fasting-induced seizure reduction; today, it stands on a robust mechanistic foundation. Ketone bodies (KBs)—principally β-hydroxybutyrate (β-HB) and acetoacetate—serve not only as alternative energy substrates but also as potent signaling metabolites. β-HB functions as a histone deacetylase inhibitor, implicating epigenetic regulation in KD’s efficacy. Furthermore, KBs support mitochondrial bioenergetics, suppress oxidative stress, modulate apoptosis, and attenuate inflammation [1]. Thus, we moved from a “weight loss diet” to a “nutritional metabolic therapy”, moving from the “ketotoxic” paradigm to the “ketohormetic” one [2]. KBs’ integration into metabolic signaling networks underscores the rationale behind KD’s expanding therapeutic applications. Beyond the extensive body of research on KD and epilepsy, recent years have seen a growing accumulation of evidence supporting the positive effects of KD and KBs on neurodegenerative disorders, cerebral ischemia, traumatic injuries, and Parkinson’s disease [3], primarily through mechanisms related to mitochondrial improvement [4], suppression of neuroinflammation [5], and regulation of microglia activity [6]. Furthermore, new frontiers have recently emerged regarding the role of KD in psychiatric disorders, including Obsessive–Compulsive Disorder [7], depressive disorders [8], and feeding and eating disorders [9]. As mentioned, obesity, type 2 diabetes mellitus (T2DM), and dyslipidemia represent domains where KD exhibits pronounced metabolic impact. Randomized trials and narrative reviews report significant weight loss, improved glycemic control (e.g., HbA1c reductions), and lipid profile amelioration in metabolic syndrome and T2DM patients [10]. On this topic, Klonek and co-workers showed that 12-week hypocaloric KD in overweight and obese women yielded substantial improvements in insulin resistance, triglycerides, HDL-C, and anthropometrics [11]. In general, KD shows moderate-to-high quality evidence for favorable cardiometabolic outcomes, though its association with elevated LDL cholesterol warrants monitoring [12].

Intriguingly, the induction of ketosis through fasting or KD has gained attention as a potential adjunctive strategy in oncology, targeting cancer metabolism through carbohydrate restriction, reduced insulin/IGF-1 signaling, and increased ketone body availability. Tumor cells often display impaired metabolic flexibility and rely heavily on glycolysis, making them potentially vulnerable to low-glucose, ketone-rich environments. Preclinical and early clinical evidence indicates that KD may enhance conventional cancer therapies such as radiotherapy and chemotherapy, by improving oxidative stress resilience in normal tissues while sensitizing tumor cells to damage. KD may also act synergistically with histone deacetylase inhibitors and influence tumor metabolomics in ways that could slow growth [13,14]. In animal models, KD has been shown to sensitize tumor cells to radiotherapy and chemotherapy by elevating oxidative stress in malignant cells, while sparing normal tissues—thus improving treatment efficacy and potentially reducing harm to healthy cells [15]. Few studies have been conducted on humans, but in a phase 1 trial, KD was shown to be safe and feasible for glioblastoma patients receiving standard of care treatment, showing potential for outcome improvement [16]. In prostate cancer, obesity is a recognized risk factor for disease progression, particularly during active surveillance. In this Special Issue a clinical study of overweight and obese men with untreated prostate cancer on active surveillance, an 8-week ad libitum KD led to a mean BMI reduction of 7.4% without adverse changes in PSA or inflammatory biomarkers, and with favorable pathological findings in a subset of patients, including tumor downgrading and remission in some cases [17]. While preliminary results are promising, especially in weight management and metabolic modulation, robust randomized trials with long-term follow-up are essential to determine KD’s role, safety, and efficacy in diverse cancer populations.

Despite its therapeutic promise, KD poses potential side effects—constipation, dyslipidemia, micronutrient imbalances, and kidney stone risk—particularly in long-term use [12]. Muscle mass loss is a concern in early phases, although data suggest preserved strength and function after the initial phase [18]. Adherence challenges also highlight the necessity for dietitian guidance and behavioral strategies.

Given interindividual variability influenced by genetics, microbiome [19], sex, age, and comorbidities, precision nutrition approaches are critical. Tailored KD variants, ranging from the classic 4:1 ratio to modified Atkins or MCT-based protocols, should align with patient preferences, clinical tolerance, and rehabilitation objectives.

To fully integrate KD into therapy and rehabilitation, the following trajectories are pivotal:

− Long-term, controlled clinical trials, particularly in neurorehabilitation and non-epilepsy populations.
− Exploration of cyclic, targeted, or adjunctive KD strategies, including ketone supplementation or time-restricted feeding.
− Mechanistic investigations across domains—mitochondrial dynamics, inflammation modulation, and neuronal repair.
− Precision KD protocols refined by genomic, metabolomic, and microbiome profiling.
− Multidisciplinary implementation models employing digital health, continuous monitoring, and collaborative therapy teams.

KD represents a paradigm shift from a niche neurological diet to a multifaceted therapeutic and rehabilitative tool. Its convergence of metabolic, anti-inflammatory, and neuroprotective mechanisms underpins its broad applicability. Realizing this potential, however, demands rigorous research, personalized protocols, and integrated care frameworks. This Special Issue of *Nutrients* showcases the breadth of KD’s emerging roles, laying the groundwork for its strategic incorporation in therapy and rehabilitation.
